# Factorial structure of the patient health questionnaire-9 in long-term survivors of severe COVID-19

**DOI:** 10.3389/fpsyg.2026.1657877

**Published:** 2026-04-24

**Authors:** Juan Carlos Rivas, Anita Restrepo, Mario Miguel Barbosa, Brayan Daniel Cordoba-Melo, Andrea Alejandra Arteaga-Tobar, Maria Camila Naranjo-Ramirez, María Corina Ochoa-Rojas, Carlos Alberto Miranda-Bastidas, Andrés Felipe Casanova Rojas, Andrés Fernando Mina Sánchez, José Rafael Tovar Cuevas, Juan Esteban Gómez-Mesa

**Affiliations:** 1 Departamento de Psiquiatría, Fundación Valle del Lili, Cali, Valle del Cauca, Colombia; 2 Facultad de Ciencias de la Salud, Universidad Icesi, Cali, Valle del Cauca, Colombia; 3 Departamento de Psiquiatría, Universidad del Valle, Cali, Valle del Cauca, Colombia; 4 Departamento de Psiquiatría, Hospital Universitario Psiquiátrico del Valle, Cali, Valle del Cauca, Colombia; 5 Centro de Investigaciones Clínicas, Fundación Valle del Lili, Cali, Valle del Cauca, Colombia; 6 Department of Psychology, University of Chicago, Chicago, IL, United States; 7 Escuela de Estadística, Universidad del Valle, Cali, Valle del Cauca, Colombia; 8 Departamento de Cardiología, Fundación Valle del Lili, Cali, Valle del Cauca, Colombia; 9 Consejo Interamericano de Falla Cardíaca e Hipertensión Pulmonar (CIFACAH) de la Sociedad Interamericana de Cardiología, Ciudad de México, Mexico

**Keywords:** depression, PHQ-9, post COVID conditions, reliability, validation

## Abstract

**Introduction:**

Depression is prevalent among survivors of severe COVID-19, yet psychometric evidence for screening instruments in this population remains limited, particularly in Latin America. The factor structure of the Patient Health Questionnaire-9 (PHQ-9) remains unclear in long-term survivors of severe COVID-19. This study aimed to evaluate the psychometric properties and factor structure of the PHQ-9 in a Colombian cohort of long-term survivors of severe COVID-19.

**Materials and methods:**

The PHQ-9 was administered to 177 individuals previously hospitalized with severe COVID-19, with follow-up extending up to 20 months post-discharge. Both exploratory (EFA) and confirmatory factor analyses (CFA) were conducted to evaluate competing structural models.

**Results:**

The unifactorial solution demonstrated superior internal consistency (*α* = 0.81, *ω* = 0.86) compared with the two-factor solution (Factor 1: α = 0.74; Factor 2: α = 0.61). The two-factor item distribution did not correspond to established depression frameworks. CFA showed both models had acceptable fit, but chi-square difference testing revealed no significant improvement for the two-factor model (Δχ^2^ = 0.27, *p* = 0.602). The fatigue item exhibited the lowest factor loading.

**Conclusion:**

The PHQ-9 demonstrated a predominantly unidimensional structure in this population. The weak performance of the fatigue item suggests potential overlap between long COVID–related fatigue and depressive symptomatology, warranting future criterion validation against structured psychiatric assessment.

## 1 Introduction

The COVID-19 pandemic has had far-reaching global consequences, with long-term sequelae continuing to affect large segments of the population worldwide. In the scientific literature, these persistent manifestations are commonly termed Long COVID (LC), post-COVID-19 condition, or post-acute sequelae of SARS-CoV-2 infection (Shah et al., 2025). For consistency, we use the term LC throughout this manuscript.

It has been estimated that around one-quarter (23%) of patients who suffered from COVID-19 had symptoms of depression during follow-up (Seighali et al., 2024), with evidence indicating that these symptoms may persist in up to 18% of individuals 2 years after SARS-CoV-2 infection (Fernandez-de-las-Peñas et al., 2024). The mechanisms underlying LC and its neuropsychiatric manifestations remain incompletely understood. The relative contribution of biological (Mazza et al., 2022; Raijmakers et al., 2025; Shankar et al., 2025), psychological (Bourmistrova et al., 2022; Selvakumar et al., 2025), and social and sociodemographic factors (Shah et al., 2025; Tebeka et al., 2023; D’Hondt et al., 2024; Delpierre et al., 2025) has not been clearly established. Taken together, these observations support a broader biopsychosocial understanding of depressive symptoms in LC, with potentially different drivers across populations and severity strata (Raijmakers et al., 2025; Selvakumar et al., 2025; Delpierre et al., 2025). For these reasons, it is essential to further characterize depressive symptoms during follow-up in populations who suffered severe COVID-19.

Many different scales have been developed to measure self-reported symptoms of depression without requiring a clinical diagnosis. Commonly used scales include the Center for Epidemiologic Studies – Depression scale (CES-D; Radloff, 1977), the Hamilton Depression Rating scale (Hamilton, 1960), the Beck Depression Inventory (BDI; Beck et al., 1961), and the Patient Health Questionnaire-9 (PHQ-9; Kroenke et al., 2001). Nonetheless, different scales can vary in their performance even within the same population (Uher et al., 2008). Among these instruments, the PHQ-9 is the most commonly used measure for assessing depression in patients with COVID-19, including studies with follow-up periods extending up to 3 years after the acute infection (D’Hondt et al., 2024; Lee et al., 2021; Bota et al., 2024). However, it remains unclear whether the PHQ-9 behaves similarly in individuals with a history of severe COVID-19 and LC as it does in the general population.

The factor structure of the PHQ-9 has been evaluated across multiple populations, with inconsistent findings. Some studies support a single-factor structure (González-Blanch et al., 2018), whereas others favor a two-factor solution generally composed of “Somatic” and “Cognitive/Affective” domains (Richardson and Richards, 2008; Elhai et al., 2012). However, even when a two-factor model demonstrates better statistical fit, the correlation between factors is often high, suggesting that a unidimensional structure may remain preferable for practical interpretation (Boothroyd et al., 2019). Therefore, the factorial structure of the PHQ-9 may vary according to the population being studied.

In Latin America, systematic psychometric evaluations of the PHQ-9 scale for diagnosing depression within the context of LC have not been reported. Latin American countries comprise culturally and ethnically diverse populations and face structural challenges, including health-system fragmentation and marked social inequities, which may influence both access to mental health care and the context in which screening tools are applied (Ruano et al., 2021; Yepes and Marín, 2018).

In addition, depressive symptoms in LC may vary according to clinical severity, and their underlying determinants remain incompletely understood (Raijmakers et al., 2025; Selvakumar et al., 2025; Delpierre et al., 2025; Weiß et al., 2024). Therefore, context-specific validation of screening instruments is needed in the region. This study aimed to evaluate the psychometric properties of the PHQ-9 in long-term survivors of severe COVID-19 from a Colombian tertiary care center, providing initial evidence to support more appropriate interpretation of PHQ-9 scores during LC follow-up in Latin American clinical settings.

## 2 Methods

### 2.1 Study design and participants

The patients analyzed were part of a prospective multicenter study that utilized data from the Latin American Registry of Cardiovascular Disease and COVID-19 (CARDIO COVID 19–20) (Gomez-Mesa et al., 2023). This registry included 3,260 patients from 43 Latin American institutions, those patients were at least 18 years old and to have been hospitalized for COVID-19 between May 2020 and June 2021. Out of these patients, 1,980 were categorized as suffering from severe COVID-19, and 1,626 patients were alive at the 30-day follow-up after hospital discharge.

Patients were classified as having severe COVID-19 if they displayed myocardial injury (elevated troponin), a high risk of venous thromboembolism (elevated D-dimer), experienced cardiovascular complications during hospitalization (such as arrhythmia, thromboembolism, coronary events, and heart failure), or required intensive care unit (ICU) treatment.

After completing the CARDIO COVID 19–20 registry, the CARDIO COVID 20–21 registry was established with the goal of monitoring all patients who had severe COVID-19 as part of the original registry. A total of six institutions accepted to participate in this project. Among them, 514 of 1,626 patients from the initial registry were identified as still alive at the 30-day follow-up in the six participant institutions (275 from Fundación Valle del Lili, Colombia). We successfully completed a follow-up assessment in 272 of those patients (177 from Fundación Valle del Lili, Colombia). These assessments were conducted either in person or virtually and occurred more than 20 months after hospital discharge, spanning from February 2022 to February 2023. The population under consideration comprised 177 patients from Colombia who completed all required psychiatric assessments during in-person (face-to-face) follow-up evaluations.

Data on demographic variables, comorbidities, signs and symptoms, physical examinations, lifestyle, and cardiovascular complications were collected. Various psychiatric assessment scales were applied: Generalized Anxiety Disorder [GAD-7, (anxiety)], PHQ-9 (depression), Perceived Stress Scale [PSS, (perceived stress)], European Quality of Life-5 Dimensions [EQ-5D, (quality of life)], and Addenbrooke’s Cognitive Examination [ACE, (cognitive impairment)]. Additionally, sub-studies involving serological tests (troponin, Pro-B-type natriuretic peptide [Pro-BNP], complete blood count, D-dimer, and serum creatinine) and imaging tests (transthoracic echocardiogram and/or cardiac magnetic resonance imaging) were conducted. All clinical, laboratory, imaging, and psychiatric data were systematically recorded and managed using the REDCap (Research Electronic Data Capture) electronic database system.

The current study focused on the subset of the sample who had completed all five psychiatric assessment scales but specifically on the PHQ-9. The PHQ-9 comprises nine questions graded on a Likert scale from 0 to 3, resulting in a total score ranging from 0 to 27. Depression severity was classified as none (0–4 points), mild (5–9 points), moderate (10–14 points), fairly severe (15–19 points), and severe (>20 points; Kroenke et al., 2001).

A PHQ-9 cutoff score of ≥10 has been shown to yield sensitivity and specificity of approximately 88% for major depressive disorder (Kroenke et al., 2001). Lower cutoff points have also demonstrated acceptable screening performance, with meta-analytic evidence supporting thresholds between 8 and 11 and local validation data indicating high sensitivity at ≥7 (Manea et al., 2012; Cassiani-Miranda et al., 2021). Because the present study was not designed as a diagnostic accuracy study, results are presented using both ≥7 and ≥10 cutoff points to facilitate interpretation across screening- and severity-based frameworks.

This study aimed to explore the factor structure of the PHQ-9 in a sample of 177 patients who were previously hospitalized due to COVID-19 in Cali, Colombia. Depression symptoms were assessed 20 months post-hospital discharge. Specifically, single-factor and dual-factor structures were investigated, as previous research on a culturally similar sample from the general population supported a two-factor model with somatic and non-somatic domains (Miranda and Scoppetta, 2018).

### 2.2 Statistical analysis

The descriptive analysis was conducted as follows: Qualitative variables were evaluated by assessing their frequency and percentage, while quantitative variables were studied by analyzing their mean and standard deviation (*SD*), as well as median with interquartile range. The Chi-square test and exact Fisher’s test were used to compare variables by sex. Kruskal-Wallis tests were employed to compare continuous variables.

**Figure 1:**
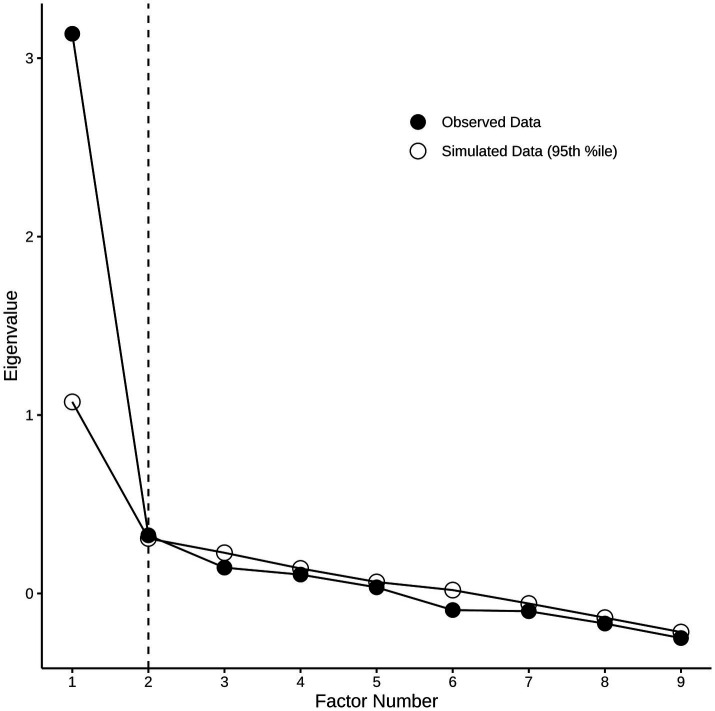
Parallel analysis scree plot for factor retention in the PHQ-9.

To explore the dimensional structure of the PHQ-9, exploratory factor analysis (EFA) was conducted using principal axis factoring applied to the correlation matrix, with varimax (orthogonal) rotation. The number of factors to retain was determined through parallel analysis with 1,000 simulated datasets, comparing observed eigenvalues to those expected by chance (Figure 1). Items with factor loadings ≥0.35 were considered salient indicators of their respective factors. Subsequently, two competing structural models were evaluated using CFA with robust maximum likelihood estimation (Satorra and Bentler, 1994). The first model specified a unifactorial depression structure with all nine items loading on a single factor, while the second model specified a two-factor structure distinguishing Somatic and Affective- Cognitive dimensions as proposed in medical populations (Kroenke et al., 2010). No rotation was applied in CFA, as factor structures were specified *a priori* based on theoretical considerations (Matsunaga, 2010).

For the Root Mean Square Error of Approximation (RMSEA), a value of 0.08 indicated good fit. For the Comparative Fit Index (CFI), an acceptable fit was considered if the value was at least 0.9. For the Standardized Root Mean Square Residual (SRMR), a value of 0.08 or less indicated good fit. Additionally, we reported Akaike Information Criterion (AIC) and Bayesian Information Criterion (BIC) scores to evaluate the fit of models. Model fit was assessed using CFI and Tucker–Lewis Index (TLI) (≥0.95 excellent, ≥0.90 acceptable), RMSEA (≤0.06 excellent, ≤0.08 acceptable), and SRMR (≤0.08 acceptable; Hu and Bentler, 1999). Because the models were nested, they were formally compared using the Satorra-Bentler scaled chi-square difference test, which adjusts for the use of maximum likelihood robust (MLR) estimation.

To further evaluate the robustness of the factorial solution, two additional two-factor models based on published item specifications were tested: the Boothroyd/Elhai specification (Elhai et al., 2012; Boothroyd et al., 2019) and the Beard specification (Beard et al., 2016). All three two-factor models were compared against the unifactorial model using scaled chi-square difference tests.

Varimax rotation was initially selected to maximize interpretability and consistency with prior exploratory analyses of the PHQ-9. However, because somatic and cognitive-affective dimensions of depression are often correlated, a sensitivity analysis was also performed comparing the varimax solution with oblique rotations (promax and oblimin).

A non-significant chi-square difference (*p* > 0.05) would indicate that the more complex two-factor model does not provide significantly better fit than the simpler unifactorial model. Differences in fit indices (ΔCFI, ΔRMSEA) and information criteria (ΔAIC, ΔBIC) were examined as supplementary evidence, with smaller values favoring the more parsimonious model. Reliability was assessed via Cronbach’s alpha and McDonald’s omega (≥0.80 good). All analyses were performed using R Statistical Software (version 4.02; RStudio Desktop, 2026; R: the R project for statistical computing, 2026) and EFA and CFA were implemented with the lavaan package (Rosseel, 2012).

### 2.3 Sample size

Sample size adequacy for CFA was evaluated *post hoc* using the Simulation-Enabled Estimation of Necessary Sample size for Exploratory and Confirmatory Analyses (SENECA) method (Lorenzo-Seva and Ferrando, 2024). SENECA estimates the minimum required sample size based on the number of observed variables, the hypothesized factor structure, and the empirical levels of communality, using Monte Carlo simulations calibrated to a prespecified fit criterion. In this study, adequacy was determined using a Correlation Root Mean Square Residual (CRMSR) threshold of 0.05, consistent with recommendations for acceptable model fit in structural equation modeling.

## 3 Results

A total of 177 patients were included in the analysis. The majority of patients were men (58%), with a median age of 57 years (IQR 47–66). Regarding medical history, the most frequent comorbidities were overweight or obesity (49%), arterial hypertension (42%), and diabetes mellitus (17%). During the acute COVID-19 episode, 102 patients (57.6%) required ICU admission. Most participants lived in urban areas (89.8%) and had a median of 11.0 years of schooling (IQR 6.0–14.3; Supplementary Table S1). Demographic and clinical characteristics are presented in Table 1.

**Table 1:** Baseline clinical and demographics characteristics.

Variable	n	Overall, *n* = 177^a^	Female, *n* = 75^a^	Male, *n* = 102^a^	*p*-value^b^
Age	177	57 (47, 66)	57 (41, 66)	57 (48, 66)	0.3
Comorbidities	177				
Overweight/Obesity	173	85 (49%)	37 (50%)	48 (48%)	0.9
Arterial Hypertension	176	74 (42%)	31 (41%)	43 (43%)	0.9
Diabetes Mellitus	176	30 (17%)	11 (15%)	19 (19%)	0.5
ICU admission	177	102 (58%)	35 (47%)	67 (66%)	0.014
Days in ICU	102	9 (5, 18)	8 (3, 20)	10 (6, 17)	0.4
PHQ-9 Total Score	177	4 (1, 9)	6 (2, 12)	3 (1, 7)	0.001
PHQ-9 Severity Categories	177				0.003
Healthy (0–4)		90 (51%)	30 (40%)	60 (59%)	
Mild (5–9)		53 (30%)	23 (31%)	30 (29%)	
Moderate (10–14)		18 (10%)	10 (13%)	8 (7.8%)	
Moderate–Severe (15–19)		14 (7.9%)	12 (16%)	2 (2.0%)	
Severe (>20)		2 (1.1%)	0 (0%)	2 (2.0%)	
PHQ-9 Clinically Relevant Cut-offs	177				
Score ≥ 7		65 (37%)	36 (48%)	29 (28%)	0.012
Score ≥ 10		34 (19%)	22 (29%)	12 (12%)	0.006

a Median (IQR); n (%).

b Wilcoxon rank sum test; Fisher’s Exact Test for Count Data; Fisher’s Exact. Test for Count Data with simulated *p*-value (based on 2000 replicates).

ICU, Intensive Care Unit; PHQ-9, Patient Health Questionnaire-9.

The median PHQ-9 score was 4.0 (IQR 1.0–9.0). Using a cutoff score of ≥7, 65 patients (37%) screened positive for depressive symptoms, whereas applying the more conservative cutoff of ≥10, 34 patients (19%) screened positive. Females were more likely than males to screen positive at both thresholds (≥7: 48% vs. 28%, *p* = 0.012; ≥10: 29% vs. 12%, *p* = 0.006; see Table 1). The mean and standard deviation of responses to each item in the PHQ-9 are presented in Table 2. Skewness coefficients ranged from 0.57 to 2.51, while kurtosis coefficients ranged from 0.08 to 5.33. Most items demonstrated adequate item–total correlations, while the fatigue item exhibited the lowest item–total correlation, and the internal consistency estimate was highest when this item was removed.

**Table 2:** Item-level descriptive statistics and reliability measures for the patient health questionnaire-9 (PHQ-9).

Item	Not at all	Several days	More than half the days	Nearly every day	Cronbach’s alpha if item deleted	Item-total correlation
1. Anhedonia	116 (66%)	54 (31%)	4 (2.3%)	3 (1.7%)	0.78	0.62
2. Depressed Mood	110 (62%)	33 (19%)	29 (16%)	5 (2.8%)	0.76	0.80
3. Sleep Disturbance	80 (45%)	37 (21%)	23 (13%)	37 (21%)	0.77	0.69
4. Fatigue	81 (46%)	53 (30%)	26 (15%)	17 (9.6%)	0.81	0.4
5. Appetite Changes	113 (64%)	29 (16%)	26 (15%)	9 (5.1%)	0.79	0.51
6. Low Self-Esteem	119 (67%)	26 (15%)	15 (8.5%)	17 (9.6%)	0.80	0.5
7. Concentration Difficulties	116 (66%)	32 (18%)	14 (7.9%)	15 (8.5%)	0.79	0.5
8. Psychomotor Disturbances	113 (64%)	25 (14%)	25 (14%)	14 (7.9%)	0.79	0.48
9. Suicidal Ideation	147 (83%)	13 (7.3%)	10 (5.6%)	7 (4.0%)	0.78	0.64

The Kaiser–Meyer–Olkin (KMO) measure yielded a robust value of 0.85, indicating substantial sampling adequacy for EFA. Bartlett’s test of sphericity was significant (χ^2^ = 434.63, df = 36, *p* < 0.001), further supporting the suitability of the data for factor analysis. In the EFA, depression was initially considered as a unidimensional construct. Scree plot inspection and parallel analysis supported up to two factors, as illustrated in Figure 1. Accordingly, both one-factor and two-factor EFA solutions were examined. In the EFA, the single-factor solution showed better internal reliability than the two-factor solution, with higher Cronbach’s alpha (*α* = 0.81) and McDonald’s omega (*ω* = 0.86) for the unifactorial solution, compared with lower reliability estimates for the two-factor solution (Table 3).

**Table 3:** Exploratory and confirmatory factor loadings and communalities for the PHQ-9 items.

Item	One-factor loading	Two-factor loading: Factor 1	Two-factor loading: Factor 2	CFA standardized loading	Communality (1F)	Communality (2F)
Depressed mood	0.83	**0.63**	0.53	0.85	0.72	0.70
Sleep disturbances	0.70	0.47	**0.52**	0.72	0.51	0.52
Suicidal ideation	0.65	**0.71**	0.20	0.63	0.40	0.53
Anhedonia	0.62	0.37	**0.53**	0.63	0.40	0.43
Appetite changes	0.51	**0.49**	0.26	0.51	0.26	0.27
Low self-esteem	0.50	**0.61**	0.08	0.50	0.25	0.38
Concentration difficulties	0.50	0.30	**0.42**	0.49	0.24	0.26
Psychomotor disturbances	0.48	**0.37**	0.30	0.46	0.21	0.22
Fatigue	0.39	0.02	**0.61**	0.38	0.14	0.31

Items are ordered according to their loading in the one-factor solution. Bold values indicate the dominant loading in the two-factor solution. 1F, one-factor solution; 2F, two-factor solution; CFA, confirmatory factor analysis.

Examination of the two-factor exploratory solution showed that items with the highest loadings on the first factor were Depressed Mood, Appetite Changes, Low Self-Esteem, Psychomotor Disturbances, and Suicidal Ideation, whereas the second factor included Anhedonia, Sleep Disturbances, Fatigue, and Concentration Difficulties. This item distribution does not correspond to previously described two-factor structures of the PHQ-9 and does not reflect a coherent theoretical separation between somatic and affective or cognitive symptoms.

Based on these exploratory findings, CFA was conducted to formally compare a theory-driven unifactorial model with a two-factor somatic/affective–cognitive model. Both models demonstrated acceptable fit indices (Table 4). However, chi-square difference testing revealed no significant improvement in fit for the two-factor model compared with the unifactorial model (Δχ^2^ = 0.27, *p* = 0.602). To assess whether the choice of item specification influenced these conclusions, two additional two-factor models based on published assignments were evaluated (Table 4). Neither the Boothroyd/Elhai nor the Beard specification showed significant improvement over the unifactorial model (Δχ^2^ = 0.181, *p* = 0.671 and Δχ^2^ = 0.784, *p* = 0.376, respectively). Interfactor correlations across all CFA two-factor models were near-unity or inadmissible (*φ* = 0.955–1.034), indicating that somatic and cognitive-affective factors were not empirically separable in this sample, regardless of item assignment.

**Table 4:** Confirmatory factor analysis fit indices for competing PHQ-9 models in long-term survivors of severe COVID-19.

Model	χ^2^	df	*p*	CFI	TLI	RMSEA (90% CI)	SRMR	AIC	BIC	φ
Unifactorial^a^	47.59	27	0.009	0.950	0.933	0.066 (0.033–0.096)	0.049	3853.1	3938.9	—
Two-factor (own)^b^^,^^c^	47.32	26	0.007	0.948	0.928	0.068 (0.041–0.093)	0.049	3854.8	3943.8	1.034
Two-factor Boothroyd/Elhai^d^	40.96	26	0.031	0.958	0.942	0.061 (0.017–0.095)	0.049	3854.8	3943.8	0.974
Two-factor Beard^e^	40.12	26	0.038	0.960	0.944	0.059 (0.013–0.094)	0.048	3854.0	3942.9	0.955

a All PHQ-9 items loading onto a single factor.

b Somatic factor: Anhedonia, Sleep disturbance, Fatigue, and Appetite Changes; affective-cognitive factor: Depressed Mood, Low Self-Esteem, Concentration Difficulties, Psychomotor Disturbances, and Suicidal Ideation.

c φ > 1.0 indicates an inadmissible solution.

d Somatic factor: Sleep disturbance, Fatigue, Appetite Changes, and Psychomotor Disturbances; cognitive-affective factor: Anhedonia, Depressed Mood, Low Self-Esteem, Concentration Difficulties, and Suicidal Ideation.

e Somatic factor: Sleep disturbance, Fatigue, Appetite Changes, Concentration Difficulties, and Psychomotor Disturbances; cognitive-affective factor: Anhedonia, Depressed Mood, Low Self-Esteem, and Suicidal Ideation.

AIC, Akaike Information Criterion; BIC, Bayesian Information Criterion; CFI, Comparative Fit Index; RMSEA, Root Mean Square Error of Approximation; SRMR, Standardized Root Mean Square Residual; TLI, Tucker–Lewis Index; φ, interfactor correlation.

As a sensitivity analysis, the two-factor EFA solution was re-estimated using oblique rotations (promax and oblimin). These yielded interfactor correlations of φ = 0.69 and φ = 0.58, respectively; explained variance and factor loadings were similar across rotation methods (Supplementary Table S2), supporting the unidimensional interpretation.

**Figure 2:**
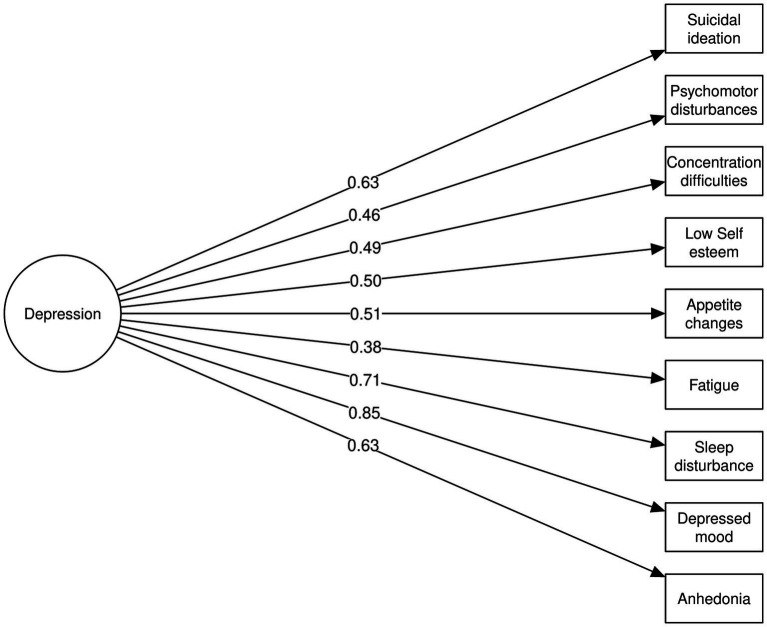
Confirmatory factor analysis path diagram for the unifactorial PHQ-9 model.

Accordingly, given the higher internal consistency of the unifactorial solution and the lack of theoretical coherence of the two-factor structure, the single-factor model was considered more appropriate for the current sample. The CFA for the unifactorial model demonstrated adequate fit, as shown in Figure 2 and Table 4. *Post-hoc* power analysis indicated that the sample size exceeded the minimum required (*N* = 155) for a one-factor model with a mean communality of 0.35. Data suitability was further supported by adequate sampling adequacy and item communality estimates (KMO = 0.854; Bartlett’s test *p* < 0.001; mean communality = 0.35, range: 0.14–0.72). Consistent with the lower item–total correlation observed in the EFA, the fatigue item also exhibited the lowest standardized loading in the confirmatory model.

## 4 Discussion

The current study sought to describe the factor structure of the commonly used PHQ-9 in a sample of patients with previous severe COVID-19 evaluated more than 20 months after the acute episode. A considerable proportion of patients reported depressive symptoms at long-term follow-up, varying according to the PHQ-9 cut-off applied, although it remains unclear whether these symptoms represent a manifestation of LC, reflect pre-existing vulnerability, or arise from bidirectional interactions between mental health and LC symptoms (Tebeka et al., 2023). EFA and CFA demonstrated that, while both one- and two-factor structures adequately fit the data, the single-factor structure was more internally consistent. Additionally, the pattern of item loadings for the two-factor model did not align with any theoretical frameworks of depression. For these reasons, we deem a single-factor structure of the PHQ-9 as the most adequate representation of the scale’s latent construct for this population.

This study adds to the growing body of evidence suggesting that the factor structure of depression scales, particularly the PHQ-9, may vary across populations and clinical contexts. In Colombia, depression often remains underdiagnosed because of persistent stigma surrounding mental health conditions and because many patients initially consult primary care or family physicians with limited training in mental health screening (Cassiani-Miranda et al., 2021). Access to formal psychiatric assessment may be further restricted among individuals from rural settings or with lower educational attainment and socioeconomic status (Yepes and Marín, 2018). Consequently, many patients are evaluated through brief screening instruments rather than structured psychiatric interviews. Under these circumstances, characterizing the factorial structure and psychometric performance of the PHQ-9 becomes especially relevant, since cultural and structural factors may influence both symptom expression and the way depressive symptoms are captured by the scale.

While some studies have found support for similar two-factor structures in distinct populations (e.g., National Guard veterans and patients with spinal cord injury; Richardson and Richards, 2008; Elhai et al., 2012), others have reported that the PHQ-9 is better represented as a unidimensional construct in both community and clinical samples (González-Blanch et al., 2018; Boothroyd et al., 2019; Titov et al., 2011). Our findings support the latter interpretation. Regardless of the item specification tested, the somatic and cognitive-affective dimensions were highly overlapping, with near-unity interfactor correlations across CFA models and moderate correlations in the oblique EFA sensitivity analyses. This pattern is consistent with prior PHQ-9 research showing that, even when two-factor models achieve slightly better statistical fit, the magnitude of the correlation between factors supports treating the instrument as essentially unidimensional (Boothroyd et al., 2019). Thus, although a somatic versus cognitive-affective distinction may be statistically identifiable in some settings, our results suggest that these domains do not represent sufficiently distinct constructs in long-term survivors of severe COVID-19.

As the current study did not directly test how specific characteristics of the population contribute to differences in factor structure, future research should evaluate psychological, social, contextual, immunological, and inflammatory factors that may influence depressive symptom expression in LC (Raijmakers et al., 2025; Selvakumar et al., 2025; Delpierre et al., 2025).

We found that the magnitude of the relationship between the singular Fatigue item and the rest of the scale was lower in this sample compared to other studies that implemented single-factor structures. The factor loading for this item was much weaker than those of the other items and the internal consistency of the scale was highest when the fatigue item was removed. This is in contrast to other studies with single-factor structures where the Fatigue item demonstrated equal or higher factor loadings than most other items on the scale (González-Blanch et al., 2018; Boothroyd et al., 2019).

These findings suggest that, in long-term survivors of severe COVID-19, fatigue may behave differently from core depressive symptoms as captured by the PHQ-9. One possible explanation is that fatigue represents a prominent and persistent feature of the LC clinical spectrum, particularly among survivors of critical illness and ICU admission, where it has been reported to persist for up to 20 months after acute infection (Bourmistrova et al., 2022; Legler et al., 2023; Navarra-Ventura et al., 2024; Egger et al., 2024), potentially leading to symptom overlap between LC and depression. In this context, the lower loading of item 4 may indicate that fatigue reflects processes beyond depressive symptomatology alone. Nevertheless, fatigue was assessed only through item 4 of the PHQ-9. No multidimensional instrument was available to further characterize this manifestation, and there is currently no gold-standard tool for the assessment of fatigue in LC, with prior studies using heterogeneous instruments such as the Chalder Fatigue Questionnaire, Fatigue Severity Scale, and Multidimensional Fatigue Inventory (Thomas et al., 2024). Therefore, it was not possible to determine whether the observed finding was primarily driven by persistent manifestations of LC, depressive symptoms, physical deconditioning, cardiopulmonary sequelae, or the overlap among these mechanisms.

As with fatigue, other depressive symptoms and somatic manifestations may also overlap in long-term survivors of severe COVID-19, which may complicate the interpretation of PHQ-9 scores in this population. Future receiver operating characteristic (ROC) curve and area under the curve (AUC)-based validation against psychiatric assessment in severe COVID-19 survivors is therefore warranted to assess whether PHQ-9 cut-offs should be recalibrated or the instrument adapted when persistent LC manifestations are prominent. Such validation would require comparison against an accepted diagnostic gold standard based on structured clinical psychiatric evaluation using internationally recognized diagnostic classification criteria (Villarreal-Zegarra et al., 2023).

A similar measurement challenge has been described in other chronic conditions with distinct underlying disease trajectories, such as multiple sclerosis, where fatigue has been shown to intersect with, but not necessarily reflect, depressive symptom severity (Sjonnesen et al., 2012). Taken together, these observations highlight the need for future studies to further examine how disease-specific symptom profiles influence the measurement properties of depression scales, particularly in conditions characterized by persistent fatigue.

## 5 Limitations

Several limitations should be acknowledged. First, although the primary aim of this study was to evaluate the factorial structure of the PHQ-9 rather than its diagnostic accuracy, the absence of a structured clinical psychiatric evaluation as a diagnostic gold standard limits the assessment of the diagnostic accuracy of the PHQ-9 in this population. As a result, ROC/AUC-based validation could not be performed, precluding the derivation of population-specific cutoff points or recalibration of the instrument. This also constrains the interpretation of prevalence estimates derived from different PHQ-9 thresholds, as cutoff selection could not be empirically optimized against standardized diagnostic criteria.

Second, factorial invariance across sex was not evaluated in the present study. Although women in the sample had significantly higher rates of depressive symptoms at both screening thresholds (≥7: 48% vs. 28%, *p* = 0.012; ≥10: 29% vs. 12%, *p* = 0.006), multi-group confirmatory factor analysis requires adequate sample sizes within each subgroup to yield stable and interpretable solutions. With a total sample of 177 participants, stratification by sex would have resulted in approximately 75 women and 102 men, which may be insufficient for stable multi-group CFA solutions, particularly given the mean communality observed in this sample (h^2^ = 0.35). Therefore, factorial invariance testing should be addressed in future studies with larger cohorts, in which configural, metric, and scalar invariance can be examined with sufficient statistical power.

Third, this was a single-center study that included only survivors of severe COVID-19 who could be contacted and agreed to attend the in-person follow-up assessment. This may have introduced survivorship and participation biases, since patients with the most severe disease may have died before follow-up, whereas those who declined participation or were lost to follow-up may have differed systematically from participants. In addition, surveillance bias is possible, as patients with persistent symptoms or greater healthcare engagement may have been more likely to attend the evaluation. Therefore, the observed psychometric properties and symptom distribution may not be fully generalizable to all survivors of severe COVID-19 or to other clinical settings.

Fourth, the relatively modest sample size may limit the stability and replicability of the factor solutions, despite *post-hoc* analyses indicating adequate sample size for the tested unifactorial model. Replication in larger and independent cohorts of patients with severe COVID-19 and LC is therefore warranted to confirm the robustness of the observed factor structure. In particular, the available sample may have been insufficient to detect small but potentially meaningful differences between the one- and two-factor models.

Finally, the lack of baseline (pre-COVID-19 or acute-phase) psychiatric assessments precludes evaluation of symptom trajectories over time and limits causal inference regarding the emergence or persistence of depressive symptoms in LC. The long interval between acute infection and follow-up also raises the possibility that factors unrelated to the initial illness or hospitalization may have contributed to the observed symptom patterns.

## 6 Conclusion

In this single-center Colombian cohort of long-term survivors of severe COVID-19 evaluated >20 months after the acute episode, the PHQ-9 demonstrated a predominantly unidimensional latent structure. Although one- and two-factor solutions showed acceptable fit, the unifactorial model was more internally consistent and offered a more parsimonious representation of depressive symptoms in this population. The fatigue item showed the weakest loading, suggesting potential overlap between LC-related fatigue and depressive symptomatology during long-term follow-up. These findings provide context-specific psychometric evidence to support interpretation of PHQ-9 scores in LC follow-up in Colombia and highlight the need for future criterion validation against structured psychiatric assessment.

## Data Availability

The data that support the findings of this study are not openly available due to reasons of sensitivity and are available from the corresponding author upon reasonable request.
