# Supplementary material for: Factorial structure of the patient health questionnaire-9 in long-term survivors of severe COVID-19
Source: Front Psychol. 2026 Apr 24;17:1657877. doi: 10.3389/fpsyg.2026.1657877 (PMC13157674; doi:10.3389/fpsyg.2026.1657877)
Supplement: Supplementary file 1 [file Table_1.docx]

**Table S1. Additional clinical and sociodemographic characteristics according to sex**

| **Variable** | **n** | **Overall, n = 177ᵃ** | **Female, n = 75ᵃ** | **Male, n = 102ᵃ** | **p-valueᵇ** |
| --- | --- | --- | --- | --- | --- |
| Area of residence | 177 |  |  |  | 0.8 |
| Urban |  | 159 (89.8%) | 68 (90.7%) | 91 (89.2%) |  |
| Rural |  | 18 (10.2%) | 7 (9.3%) | 11 (10.8%) |  |
| Years of education | 177 | 11.0 (6.0, 14.3) | 11.0 (7.3, 14.0) | 11.0 (6.0, 15.5) | >0.9 |
| Autoimmune Disease / Immunosuppressant Use | 176 | 20 (11%) | 9 (12%) | 11 (10.8%) | 0.8 |
| Solid Organ Transplant | 176 | 19 (10.8%) | 9 (12.3%) | 10 (9.8%) | 0.7 |
| CKD | 176 | 17 (9.7%) | 8 (10.7%) | 9 (8.8%) | 0.8 |
| Dyslipidemia | 175 | 13 (7.4%) | 3 (4.1%) | 10 (9.8%) | 0.2 |
| Asthma/COPD | 176 | 11 (6.3%) | 5 (6.7%) | 6 (5.9%) | 0.8 |
| Healthcare Worker /  Health Personnel | 177 | 13 (7.3%) | 5 (6.7%) | 8 (7.8%) | >0.9 |
| Work sector | 177 |  |  |  | <0.001 |
| Commercial or  Service Sector |  | 63 (36%) | 26 (35%) | 37 (36%) |  |
| Unpaid Work |  | 51 (29%) | 34 (45%) | 17 (17%) |  |
| Pensioner |  | 17 (9.6%) | 7 (9.3%) | 10 (9.8%) |  |
| Transportation Sector |  | 13 (7.3%) | 1 (1.3%) | 12 (12%) |  |
| Defense and Security Sector |  | 6 (3.4%) | 1 (1.3%) | 5 (4.9%) |  |
| Agricultural Sector |  | 5 (2.8%) | 1 (1.3%) | 4 (3.9%) |  |
| Laterality  (Hand Used for Writing) | 177 |  |  |  | >0.9 |
| Left |  | 8 (4.5%) | 3 (4.0%) | 5 (4.9%) |  |
| Right |  | 165 (93%) | 70 (93%) | 95 (93%) |  |
| Ambidextrous |  | 2 (1.1%) | 1 (1.3%) | 1 (1.0%) |  |

*^a^n (%).*

*^b^Wilcoxon rank sum test for continuous variables; Pearson’s chi-squared test or Fisher’s exact test for categorical variables, as appropriate.*

*Abbreviations: CKD, chronic kidney disease; COPD, chronic obstructive pulmonary disease.*

**Table S2. Fit Indices for Competing EFA Solutions According to Number of Factors and Rotation Method**

| **Model** | **Rotation** | **RMSEA** | **TLI** | **BIC** | **RMSR** | **Variance (%)** | **Mean h²** |
| --- | --- | --- | --- | --- | --- | --- | --- |
| 1F-Varimax | Orthogonal | 0.06 | 0.94 | −93.6 | 0.06 | 34.8 | 0.35 |
| 1F-Promax | Oblique | 0.06 | 0.94 | −93.6 | 0.06 | 34.8 | 0.35 |
| 1F-Oblimin | Oblique | 0.06 | 0.94 | −93.6 | 0.06 | 34.8 | 0.35 |
| 2F-Varimax | Orthogonal | 0.04 | 0.97 | −72.7 | 0.04 | 40.1 | 0.40 |
| 2F-Promax | Oblique | 0.04 | 0.97 | −72.7 | 0.04 | 40.1 | 0.40 |
| 2F-Oblimin | Oblique | 0.04 | 0.97 | −72.7 | 0.04 | 40.1 | 0.40 |

*Reference thresholds: RMSEA ≤0.05 excellent, ≤0.08 acceptable; TLI ≥0.95 excellent, ≥0.90 acceptable; BIC: lower = better relative fit; RMSR ≤0.05 excellent, ≤0.08 acceptable.*

*Abbreviations: EFA, exploratory factor analysis; 1F, one-factor solution; 2F, two-factor solution; BIC, Bayesian Information Criterion; RMSEA, Root Mean Square Error of Approximation; RMSR, Root Mean Square Residual; TLI, Tucker–Lewis Index; h², communality.*
